# Post-Translational Modifications of NTCP: A Regulatory Nexus for Bile Acid Transport and HBV Entry

**DOI:** 10.3390/biomedicines14050978

**Published:** 2026-04-24

**Authors:** Fei Yu, Yue Zhu, Na Li, Qing Peng, Fanghang Ye, Qianlan Luo, Jiajun Xia, Xiaoyu Hu

**Affiliations:** 1 School of Clinical Medicine, Chengdu University of Traditional Chinese Medicine, Chengdu 610075, China; yufei8727@163.com (F.Y.);; 2 Department of Infectious Diseases, Zigong First People’s Hospital, Zigong 643000, China; 3 Department of Infectious Diseases, Hospital of Chengdu University of Traditional Chinese Medicine, Chengdu 610072, China

**Keywords:** sodium taurocholate cotransporting polypeptide, hepatitis B virus, post-translational modification of proteins, bile acid homeostasis

## Abstract

The sodium-taurocholate cotransporting polypeptide (NTCP) plays a critical dual role in liver function: maintaining bile acid (BA) enterohepatic circulation and acting as a receptor for the entry of hepatitis B and D viruses into hepatocytes. This review outlines the impact of various post-translational modifications (PTMs) of NTCP—including phosphorylation, oligomerization, ubiquitination, and glycosylation—on its dynamic regulatory network. These modifications coordinate the modulation of NTCP’s membrane localization, stability, conformational state, and protein interactions, precisely controlling its functions in BA uptake and viral invasion. Targeting this PTM network presents a promising strategy for next-generation therapies that selectively inhibit viral infection while preserving BA transport, overcoming the limitations of conventional inhibitors that indiscriminately disrupt virus–NTCP interactions. By synthesizing recent insights into NTCP PTM research, this article highlights its role as a central regulator of its bifunctional properties and reveals potential avenues for precision therapies in viral hepatitis, cholestasis, and related liver diseases. However, most existing evidence is derived from in vitro or cell-based models, whereas in vivo studies and clinical validation remain limited; thus, the translational feasibility of strategies targeting post-translational modifications of NTCP still requires further investigation.

## 1. Introduction

The discovery of the sodium-taurocholate cotransporting polypeptide (NTCP) was a significant breakthrough in understanding the mechanisms underlying hepatic bile acid (BA) uptake. Encoded by the SLC10A1 gene, NTCP is selectively localized to the basolateral membrane of hepatocytes, where it serves as the primary mediator of BA uptake, a role confirmed by extensive research [1]. NTCP utilizes the sodium electrochemical gradient to mediate sodium-coupled BA transport with a 2:1 stoichiometry (two Na^+^ ions per BA molecule), thereby ensuring efficient reuptake of conjugated BAs from portal blood into hepatocytes and constituting the rate-limiting step in their reclamation within the enterohepatic circulation [2]. The enterohepatic circulation of BAs constitutes a highly efficient recycling system. Following conjugation in hepatocytes, BAs are secreted into bile via the bile salt export pump (BSEP), stored in the gallbladder, and released into the intestine in response to food intake. The majority of BAs are subsequently reabsorbed in the terminal ileum by the apical sodium-dependent bile acid transporter (ASBT), transported back to the liver via the portal circulation, and ultimately taken up by NTCP, thereby completing the cycle. NTCP, as a critical transporter in maintaining BA homeostasis and enterohepatic circulation, operates in concert with other proteins such as BSEP, ASBT, and OSTα/β in the liver–gut axis. These proteins form a finely tuned network that regulates the balance between BA reabsorption and excretion [3,4]. Consequently, NTCP plays a fundamental role in systemic BA homeostasis, indirectly influencing BA-dependent processes such as cholesterol metabolism and lipid absorption.

In 2012, NTCP was identified as the functional receptor for hepatitis B virus (HBV) and hepatitis D virus (HDV), highlighting its central role in viral hepatitis pathogenesis [5,6]. This finding not only illuminated the molecular basis for HBV/HDV hepatotropism but also positioned NTCP as a promising target for antiviral therapy. Subsequent cryo-electron microscopy (cryo-EM) studies in 2022 provided structural insights into its mechanism, revealing that NTCP—a nine-transmembrane protein (TM1–TM9)—contains a binding cavity formed by TM1, TM5, and TM8. This cavity specifically interacts with the N-terminally myristoylated preS1 domain of the HBV large surface protein, with key residues like glycine 158 (G158) at the amino-terminal end of TM5 being critical for this interaction, thereby facilitating viral entry [7,8]. As such, targeted modulation of NTCP has become a promising strategy for inhibiting viral entry, driving the development of new anti-HBV/HDV agents [9,10,11]. In summary, NTCP’s dual role in BA homeostasis and HBV infection highlights its essential function in hepatic pathophysiology.

While the structural and functional properties of NTCP have been well characterized, the mechanisms underlying its precise regulation of physiological BA transport and pathological HBV infection remain a major challenge in current research. Growing evidence indicates that post-translational modifications (PTMs) form a complex and dynamic regulatory network that governs this dual functionality [12,13]. PTMs—including phosphorylation [14,15], oligomerization [16,17], ubiquitination [18], and glycosylation [19]—dynamically influence NTCP’s membrane localization, endocytosis, stability, and protein interactions, thus regulating both its transport efficiency and viral receptor activity while fine-tuning HBV infection. Under physiological conditions, PTMs carefully regulate NTCP-mediated BA uptake; in contrast, during HBV infection, specific PTMs alter NTCP’s membrane trafficking and conformation, modulating viral entry efficiency [18,20]. Therefore, PTMs not only bridge NTCP’s physiological and pathological roles at the molecular level but also represent a promising target for innovative therapeutic approaches in HBV and related liver diseases [11,21]. This review provides a comprehensive analysis of the NTCP PTM network, highlighting its role as a key molecular switch that integrates BA homeostasis and viral hepatitis.

## 2. Post-Translational Modifications of NTCP

### 2.1. Phosphorylation Modifications: Central Mechanisms Regulating NTCP Function and Membrane Abundance

Protein phosphorylation, a ubiquitous and essential PTM in eukaryotic cells, facilitates rapid and precise alterations in protein conformation, activity, stability, and subcellular localization through the reversible covalent attachment of phosphate groups to specific residues—mainly serine, threonine, and tyrosine [22]. Recent research has highlighted phosphorylation as a critical regulatory mechanism controlling the stability, membrane expression, and function of BA transporters. For instance, in the human apical sodium-dependent BA transporter (hASBT; SLC10A2), a member of the SLC10 family, phosphorylation levels have been positively correlated with BA uptake activity [23]. Similarly, the apical localization and transport function of the bile salt export pump (BSEP), an ABC transporter family member, are regulated by phosphorylation via protein kinase C alpha (PKCα) [24]. In NTCP, phosphorylation is a central regulatory mechanism, influencing both its membrane abundance and transport capacity, as well as its role in maintaining BA homeostasis and modulating HBV entry [15,20].

**Figure 1:**
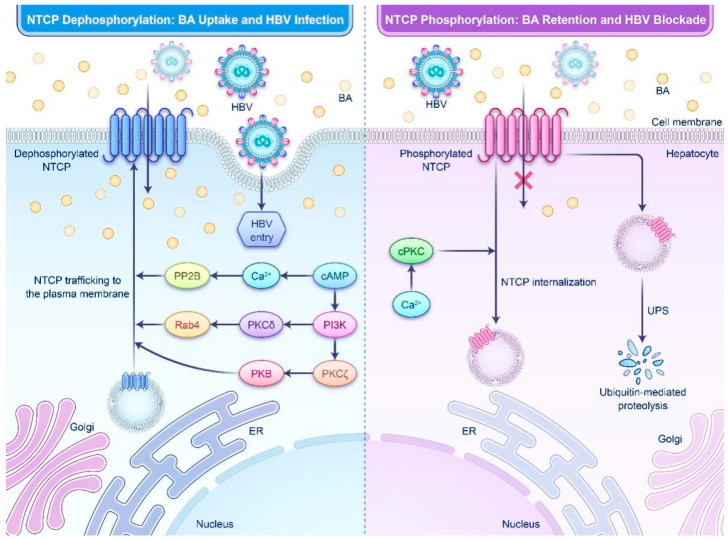
Schematic illustration of NTCP phosphorylation in regulating its membrane localization and functionality. The left panel (physiological state) depicts the stimulation of cAMP accumulation by postprandial increases in plasma BA concentrations. cAMP activates PP2B to dephosphorylate NTCP, while simultaneously engaging the PI3K/PKB pathway and downstream PKCζ (which facilitates trafficking) and PKCδ (which mediates transport via Rab4 activation). These processes collectively drive the translocation of dephosphorylated NTCP to the plasma membrane, where increased NTCP abundance enhances BA uptake but also raises susceptibility to HBV infection. The right panel (pathophysiological protective state) shows that, in conditions like acute cholestasis, high BA levels trigger intracellular Ca^2+^ signaling that activates cPKC. cPKC phosphorylates NTCP, promoting its internalization and vesicular recycling to reduce BA influx for hepatoprotection while simultaneously limiting HBV entry by decreasing surface NTCP levels. Additionally, the UPS degrades mature NTCP through the ERAD pathway. cAMP, cyclic adenosine monophosphate; PP2B, protein phosphatase 2B; PI3K, phosphoinositide 3-kinase; PKB, protein kinase B; PKCζ, protein kinase C zeta; PKCδ, protein kinase C delta; cPKC, conventional protein kinase C; UPS, ubiquitin–proteasome system; ERAD, endoplasmic reticulum-associated degradation.

Phosphorylation of NTCP serves as a key regulatory mechanism in both physiological and pathological contexts. Under normal conditions, postprandial increases in plasma BA concentrations trigger cAMP accumulation, activating calcium/calmodulin-dependent protein phosphatase 2B (PP2B). PP2B specifically dephosphorylates serine-226 (Ser-226) on NTCP, initiating its translocation to the plasma membrane [25,26]. This process is further amplified by cAMP through activation of the phosphoinositide 3-kinase (PI3K) pathway and its downstream effector, protein kinase B (PKB), which drives NTCP membrane insertion [27,28]. The orchestration of this mechanism relies on distinct protein kinase C (PKC) isoforms: PKCζ is essential for cAMP-dependent NTCP trafficking [29,30], while PKCδ facilitates this process by activating Rab4, a small GTPase involved in endosomal trafficking [28]. These kinases phosphorylate intermediary proteins that mediate vesicular transport, facilitating the shuttling of dephosphorylated NTCP from intracellular reserves to the plasma membrane (Figure 1). This cAMP-mediated regulatory network has been further confirmed in subsequent studies [31].

In pathological conditions such as acute cholestasis, elevated BA levels in hepatocytes trigger a cascade of protective responses. Initially, these high BA concentrations activate intracellular Ca^2+^ signaling, which in turn activates conventional PKC (cPKC) [32]. cPKC then promotes NTCP phosphorylation, inducing its internalization and vesicular recycling, rapidly reducing membrane abundance—a dual mechanism that both curtails BA uptake to mitigate hepatotoxicity and impedes HBV entry by decreasing surface NTCP availability [14,15]. Additionally, specific BAs, such as taurolithocholic acid (TLC), directly inhibit NTCP activity through a trans-inhibition mechanism, enhancing hepatocellular resilience against BA-induced toxicity [33].

Given that NTCP serves as the functional receptor for both HBV and HDV, its membrane abundance directly regulates viral entry efficiency. As such, signals that modulate NTCP phosphorylation and membrane trafficking—such as cAMP and BAs—can significantly influence HBV infection dynamics by altering receptor accessibility on the cell surface. For example, experimental knockdown of E-cadherin results in the aggregation of NTCP in the cytoplasm and a concomitant decrease in membrane NTCP levels, leading to a substantial reduction in HBV entry and highlighting the importance of NTCP localization in viral infection [34]. Additionally, the ubiquitin–proteasome system (UPS) mediates the degradation of mature NTCP via the endoplasmic reticulum-associated degradation (ERAD) pathway. Dysregulation of this process can lead to abnormal intracellular accumulation of NTCP, potentially contributing to the pathogenesis of cholestasis [35].

In summary, hepatocytes use reversible phosphorylation to dynamically adjust NTCP membrane positioning and functionality, thereby regulating BA uptake and HBV infectivity. Consequently, targeting NTCP phosphorylation signaling pathways offers a promising strategy for the development of novel anti-HBV therapeutics.

Currently, most evidence regarding NTCP phosphorylation is derived from studies employing pharmacological modulation of kinases or phosphatases in combination with functional readouts (e.g., transport activity and membrane localization), whereas more direct evidence—such as quantitative assessment of NTCP phosphorylation or precise mapping of phosphorylation sites—remains limited. Despite robust in vitro evidence, the in vivo dynamics of NTCP phosphorylation and its regulation by pathophysiological stimuli (e.g., cholestasis and HBV infection) remain to be directly demonstrated in human liver tissue.

### 2.2. Oligomerization Status: Structural Basis for NTCP-Mediated HBV/HDV Entry

#### 2.2.1. NTCP Oligomerization and HBV Entry

Oligomerization of membrane transporters represents a key regulatory mechanism for modulating their membrane localization and functionality. Extensive evidence shows that a variety of transporters—including the excitatory amino acid transporter 2 (EAAT2), sodium bicarbonate cotransporter NBCe1-A (NBCe1-A), GABA transporter 1 (GAT1), glycine transporter 2 (GlyT2), and ASBT—primarily function as dimers or oligomers on the plasma membrane [36,37,38,39]. Small-molecule-induced oligomerization of the dopamine transporter (DAT) can trigger transporter endocytosis, which is directly linked to DAT’s conformational dynamics [40]. Similarly, studies using rat liver tissue and U2OS cell models have shown that NTCP predominantly exists as a homodimer in its native state and is stably localized at the plasma membrane [41]. Further research identified the GXXXG/A motif within NTCP’s transmembrane domain as a critical determinant for its dimerization and membrane sorting. Mutating this motif in transmembrane domain 7 (TMD7) leads to complete loss of NTCP membrane expression and BA transport activity, as well as the abolition of HBV infectivity [42].

NTCP, a tunnel-shaped integral membrane protein with nine transmembrane helices (TM1–TM9), has an extracellular N-terminus and a cytosolic C-terminus [43]. Recent studies not only confirm NTCP as the functional receptor facilitating HBV and HDV entry into hepatocytes but also elucidate its presence on the plasma membrane as homo-oligomers, such as homodimers. This oligomerization state is critical for successful viral invasion [16]. The process of HBV entry occurs through a well-orchestrated multistep cascade, beginning with low-affinity binding between the virus and hepatocyte surface heparan sulfate proteoglycans (HSPGs) [44]. Following this, the preS1 domain of the HBV large surface protein binds to NTCP with high affinity. Structural virology analyses have revealed that the N-terminal segment of the preS1 peptide deeply embeds into NTCP’s extracellular tunnel, while its C-terminal segment establishes extensive interactions at the tunnel’s aperture, culminating in precise viral docking [43].

**Figure 2:**
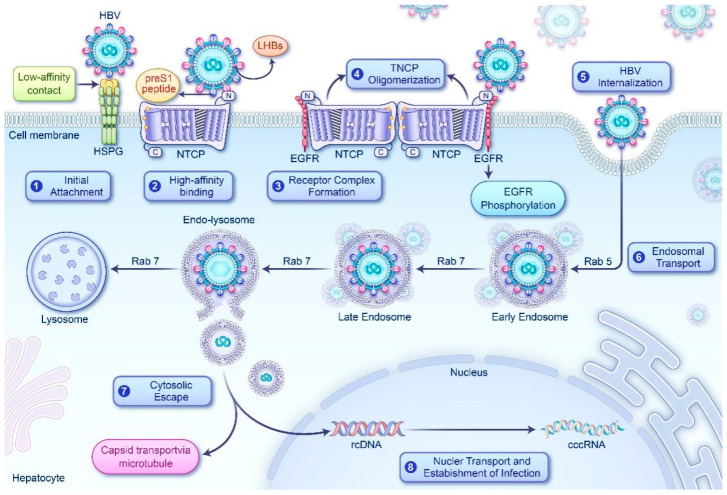
This schematic outlines the multistep process of HBV entry into hepatocytes: (1) Initial attachment: HBV virions bind to hepatocyte membrane HSPGs via low-affinity interactions with their envelope glycoproteins, anchoring to the cell surface and initiating the invasion cascade. (2) High-affinity binding: The preS1 domain of HBV large surface protein (LHBs) forms a specific high-affinity interaction with NTCP on the hepatocyte membrane. (3) Receptor complex assembly and priming: EGFR, acting as a key co-receptor, interacts with NTCP to form the NTCP–EGFR complex. HBV preS1 binding to this complex triggers downstream conformational changes. (4) NTCP oligomerization: Viral binding induces NTCP monomers to oligomerize into stable homodimers, facilitating viral internalization. (5) Internalization: Successful NTCP oligomerization activates EGFR autophosphorylation, which in turn triggers the endocytic machinery to mediate the uptake of the HBV–receptor complex into the cytosol, completing the entry process. (6) Endosomal trafficking: The virion is internalized via clathrin-mediated endocytosis, moving through early endosomes to late endosomes. (7) Cytosolic escape: Acidification within late endosomes causes capsid reconfiguration, enabling penetration of the endosomal membrane and viral release into the cytoplasm. (8) Nuclear transport and infection establishment: Cytoplasmic nucleocapsids are transported to the nuclear pore complex via microtubules, where rcDNA is released into the nucleus and converted into stable cccDNA, establishing persistent infection. EGFR, epidermal growth factor receptor; HSPGs, heparan sulfate proteoglycans; LHBs, large hepatitis B surface antigen; NTCP, sodium-taurocholate cotransporting polypeptide; rcDNA, relaxed circular DNA; cccDNA, covalently closed circular DNA; Rab5, Ras-related protein Rab-5A; Rab7, Ras-related protein Rab-7A.

In this complex cascade, the epidermal growth factor receptor (EGFR) plays a pivotal role as a co-receptor, exerting significant influence through interactions between its extracellular domain and specific residues on NTCP’s extracellular loops, notably G144 and G148 within the TM4–TM5 loop. This interaction facilitates the assembly of an EGFR–NTCP complex [45]. Viral–receptor binding goes beyond simple docking; it dynamically induces conformational changes in the receptor. The proximity ligation assay, a method for detecting protein interactions, provides compelling evidence: under basal conditions, NTCP maintains baseline spontaneous oligomerization. However, the HBV preS1 domain acts as a potent inducer, significantly enhancing NTCP oligomerization and stabilizing monomer interactions into stable dimers [16]. This induction is dependent on specific protein–protein interfaces; targeted mapping has identified NTCP residues 221–240 (within TM7) and 271–290 (spanning the TM8–TM9 extracellular loop and TM9 extracellular flank) as essential for NTCP–NTCP associations. Delivery of competitive peptides targeting these regions strongly suppresses oligomerization and reduces viral internalization efficacy [16]. The 271–290 segment is particularly critical as the core oligomerization interface [17]. Alanine-scanning mutagenesis has pinpointed specific amino acids in this region, with F274 emerging as a key determinant for NTCP oligomerization and HBV internalization [46]. Mutating this residue (F274A) completely disrupts NTCP oligomerization and blocks viral entry, though it does not affect the initial binding of the virus to NTCP [46]. Overall, virus-induced NTCP oligomerization forms a key signaling hub, leading to EGFR autophosphorylation and subsequent endocytic uptake, resulting in the internalization of the HBV–receptor complex to complete the infection process (Figure 2) [47].

HBV enters the cell via clathrin-mediated endocytosis, with its intracellular trafficking regulated by the sequential activation of small GTPases Rab5 and Rab7, which guide the virus from early to late endosomes [48,49]. The virus exploits the moderately acidic environment of late endosomes to trigger capsid conformational changes, facilitating membrane penetration and escape into the cytosol before lysosomal degradation [48]. Subsequently, the cytoplasmic nucleocapsids undergo microtubule-directed transport to the nuclear pore complex, where viral DNA is released into the nucleus for conversion into covalently closed circular DNA (cccDNA), establishing persistent infection [50]. Notably, lysosomal activators, such as Mn^2+^, interfere with this process by hyperactivating mechanistic target of rapamycin complex 1 (mTORC1), enhancing endosomal acidity, and causing premature viral degradation within late endosomes [51]. This highlights the importance of maintaining the functional integrity of the endosomal–lysosomal compartment as a critical checkpoint in HBV infection, indicating that not all internalized virions avoid lysosomal degradation.

Building on these insights, disrupting NTCP oligomerization has emerged as a promising antiviral strategy for developing new anti-HBV drugs. Experimental evidence supports that novel synthetic lithocholic acid derivatives, such as SO-145, and curcumin effectively reduce viral infection by destabilizing NTCP oligomerization [16,17]. These findings highlight the strategic value of targeting NTCP oligomerization interfaces in antiviral therapies. However, the precise regulatory role of NTCP oligomerization in its physiological BA transport function remains an unresolved question, necessitating further research to connect its physiological and pathological roles. Currently, most studies on NTCP oligomerization rely on overexpression systems or synthetic peptides; whether native NTCP oligomerization occurs in the same manner in primary human hepatocytes and whether it can be pharmacologically modulated in vivo remain to be determined.

#### 2.2.2. NTCP Oligomerization and HDV Entry

HDV, a satellite virus reliant on HBV envelope proteins, enters hepatocytes via the same NTCP-mediated mechanism as HBV. HDV particles specifically utilize HBV-derived envelope proteins, including the PreS1 domain, to bind NTCP and facilitate internalization [52]. Consequently, the NTCP oligomerization and associated endocytic processes outlined earlier are likely pivotal in HDV infection.

Recent structural studies offer direct molecular evidence supporting this shared mechanism. In 2022, cryo-EM revealed the high-resolution structure of NTCP, elucidating its dual function as both a BA transporter and a viral receptor. Notably, a pocket on the extracellular surface was identified, specifically interacting with the PreS1 domain [7,8]. Building on this, the 2024 Bulevirtide–NTCP complex structure further demonstrated that the viral PreS1 peptide is deeply embedded in NTCP’s transmembrane tunnel, with its N-terminal myristoyl group anchoring in the lipid membrane to form a “plug-like” structure that effectively prevents viral entry [53]. Recent findings also show that PreS1, an intrinsically disordered peptide, binds NTCP with high affinity via a multistep cooperative mechanism, with key residues, including Asn9, Gly12, and His17, forming the core binding interface [54]. These studies not only elucidate the molecular interaction between HDV and NTCP but also explain the efficacy of NTCP-targeting drugs in inhibiting both HBV and HDV infections.

Based on these mechanisms, antiviral strategies targeting the NTCP oligomerization interface may also prove effective against HDV. Research indicates that compounds such as troglitazone and SO-145, which disrupt NTCP oligomerization, can significantly inhibit HBV entry by interfering with the formation of functional NTCP oligomers [16,17]. Given that both HDV and HBV utilize the NTCP-dependent entry pathway, these compounds likely also possess the potential to inhibit HDV infection. Thus, targeting the NTCP oligomerization interface represents a promising approach for developing broad-spectrum therapeutics against both HBV and HDV.

### 2.3. Ubiquitination Modifications: Bidirectional Switch Regulating NTCP Endocytosis and Degradation

Ubiquitination plays a pivotal role in regulating cellular homeostasis by orchestrating the endocytosis and degradation of membrane proteins. NTCP ubiquitination is a key mechanism in its functional modulation, with lysine 340 (K340) identified as the principal ubiquitination site [18]. Ubiquitination at this position significantly affects NTCP membrane dynamics and transport efficiency: substitution of K340 with arginine (K340R) to mimic a deubiquitinated state increases membrane protein levels and bile salt uptake capacity, yet simultaneously results in an ~80% reduction in both endocytic efficiency and HBV infection facilitation [18]. This seemingly paradoxical outcome highlights a fundamental mechanism: K340 ubiquitination has little effect on NTCP membrane localization or its affinity for viral ligands but instead acts as a key signaling cue that drives the virus–receptor complex through clathrin-mediated endocytosis into the cytosol.

Notably, this mechanism mirrors regulatory processes observed in various neurotransmitter transporters. For example, PKC activation in DAT, glutamate transporter 1 (GLT1), and glycine transporters (GLYTs) triggers an initial ubiquitination event, which is essential for the subsequent initiation of clathrin-mediated endocytosis. Disruption of these ubiquitin-binding motifs via mutation effectively abolishes PKC-dependent endocytic trafficking [55,56,57,58].

Regarding degradative pathways, NTCP stability is regulated cooperatively by the UPS and lysosomal systems. Early studies have demonstrated that treatment with proteasome inhibitors (e.g., MG-132 or lactacystin) or lysosomal inhibitors (e.g., bafilomycin A1) leads to intracellular NTCP accumulation, highlighting the involvement of both degradation pathways in maintaining NTCP homeostasis [35,59]. Notably, nascent NTCP polypeptides during endoplasmic reticulum maturation are recognized by the UPS via the ERAD pathway, which serves as a critical checkpoint for protein quality control and steady-state regulation [35]. In contrast, ubiquitination at K340 primarily functions as a trigger for endocytosis of surface NTCP, facilitating internalization of the NTCP–virus complex and enabling HBV entry.

In summary, K340 ubiquitination acts as an autonomous molecular switch that precisely regulates NTCP endocytosis and its role in HBV entry. This discovery not only enhances our understanding of the interplay between BA transport and viral receptor dynamics but also opens new avenues for antiviral strategies targeting this regulatory nexus. Pharmacological agents, such as the ubiquitination inhibitor TAK-243, show promise in selectively inhibiting NTCP’s ubiquitination-dependent endocytosis, effectively reducing HBV infection while preserving BA homeostasis [18]. However, the K340 ubiquitination model is primarily derived from cell line-based studies; its physiological relevance in the human liver remains to be established.

### 2.4. Glycosylation Modifications: Pivotal Regulator of NTCP Membrane Localization and Viral Entry

Among NTCP PTMs, glycosylation plays a central role in regulating its functional dynamics and subcellular localization. The human NTCP polypeptide contains two conserved N-glycosylation sites at asparagines N5 and N11 [59]. Site-directed mutagenesis has demonstrated that these sites accommodate independent glycosylation events, resulting in variants with a single glycan moiety at either N11 (NTCP-N11Q) or N5 (NTCP-N5Q), while the double mutant (NTCP-N5,11Q) shows complete abrogation of glycosylation [59]. The mature N-glycosylated isoform provides the essential structural foundation for NTCP trafficking to the plasma membrane and its role in HBV infection [59,60].

Glycosylation, particularly the retention of at least one N-glycosylation site, is essential for stable plasma membrane expression of NTCP. Mutants that preserve a single site (NTCP-N5Q or NTCP-N11Q) maintain effective membrane localization, with only modest reductions in expression compared to wild-type NTCP (NTCP-WT). In contrast, the fully deglycosylated NTCP-N5,11Q shows a significant decrease in surface abundance [59]. These findings indicate that even a single glycan moiety is sufficient for NTCP membrane trafficking, while complete deglycosylation severely impairs this function.

Deglycosylation also markedly reduces NTCP’s BA transport efficacy. Although the NTCP-N5,11Q mutant retains some uptake capacity, its transport activity is substantially diminished compared to the wild-type NTCP [59]. Mechanistic studies reveal that treatment with the lysosomal inhibitor bafilomycin A1 leads to intracellular accumulation of this mutant, suggesting lysosomal degradation as the primary degradation route. Notably, even after accumulation, transport functionality remains irreversibly compromised [59]. These results suggest that glycosylation not only stabilizes NTCP but also maintains its proper tertiary conformation, which is essential for function.

Viral entry appears to be more dependent on NTCP glycosylation than its physiological transport function. Appelman et al. reported that even single-site mutations (NTCP-N5Q or NTCP-N11Q) markedly impair HBV infection, underscoring the importance of intact glycosylation for viral entry [59]. In contrast, Lee et al. demonstrated that non-glycosylated NTCP mutants retain the ability to mediate cell culture-derived HBV infection in HepG2 cells, suggesting that the requirement for glycosylation may be context-dependent [61]. These discrepancies may arise from differences in cell models (HepG2 vs. HepaRG), viral inocula (cell culture-derived vs. patient serum-derived HBV), or NTCP expression levels and therefore warrant further investigation. Supporting this notion, studies using human serum-differentiated Huh7.5-NTCP cells—a more physiologically relevant hepatocyte model—have shown significantly enhanced HBV infectivity, indicating that glycosylation may augment NTCP receptor functionality under near-physiological conditions [19]. However, most available data on NTCP glycosylation are derived from mutagenesis approaches or pharmacological inhibition in immortalized cell lines; further validation in primary human hepatocytes and in vivo models is required.

E-cadherin regulates HBV infection by controlling the membrane distribution of glycosylated NTCP. Mechanistically, E-cadherin selectively interacts with glycosylated NTCP to promote its plasma membrane localization, whereas suppression of E-cadherin leads to decreased surface expression and intracellular retention of NTCP [34]. Functional assays in HepG2-NTCP and primary human hepatocytes (PHHs) demonstrate that E-cadherin knockdown significantly reduces HBV 3.5 kb RNA, core protein, and hepatitis B surface antigen levels, while E-cadherin overexpression enhances viral infectivity. This highlights E-cadherin as a critical host factor for maintaining NTCP membrane localization and facilitating HBV entry [34].

### 2.5. Regulation of NTCP Function and Therapeutic Response by Host Genetic and Non-Genetic Factors

Genetic variation and expression regulation of the SLC10A1 gene can directly influence the NTCP protein sequence, PTMs, and functional activity, thereby affecting individual susceptibility to HBV/HDV infection and therapeutic responses. Amino acid substitution studies have shown that tyrosine at position 146 (Y146) of NTCP is essential for its HBV receptor function. Mutations such as Y146A or Y146E completely abolish the binding of the viral preS1 peptide to NTCP while preserving BA transport activity, highlighting that specific residues can dissociate NTCP’s physiological and pathological functions [62].

Clinical studies further suggest that chronic hepatitis D(CHD) patients with the rs17556915 TT/CC genotype have significantly higher baseline HDV RNA levels compared to those with the CT genotype. These patients are also more likely to experience virological non-response during bulevirtide therapy, indicating that NTCP genetic polymorphisms may serve as potential biomarkers for predicting treatment outcomes in HDV infection [63]. Moreover, SLC10A1 variants exhibit differential distribution across populations, implying that genetic diversity can influence drug transport function and therapeutic efficacy [64].

However, genetic variation alone does not account for all NTCP functional variability. A multi-omics analysis of 143 human liver samples revealed substantial inter-individual differences in SLC10A1 mRNA and NTCP protein levels, with variation up to 44-fold and 10.4-fold, respectively. Notably, this variability was primarily linked to non-genetic factors—such as smoking, alcohol consumption, and DNA methylation—rather than genetic variants [65].

In conclusion, SLC10A1 genetic polymorphisms, along with epigenetic and environmental factors, jointly regulate NTCP function. Further studies are needed to better understand the cooperative or interactive mechanisms through which genetic and non-genetic factors influence diverse PTMs of NTCP.

## 3. Compounds Targeting NTCP Post-Translational Modifications and Their Mechanisms of Action

Building on the regulatory mechanisms outlined above, targeting these key nodes has emerged as a promising strategy for anti-HBV therapies. A growing array of compounds and pharmaceuticals designed to modulate NTCP PTMs, thereby influencing both viral infection dynamics and BA homeostasis, has been progressively identified (Table 1). It is important to note that among the strategies summarized in Table 1, several agents—such as bulevirtide and cyclosporin A derivatives—have advanced to clinical evaluation, whereas most PTM-targeting approaches (e.g., modulation of phosphorylation, oligomerization, or ubiquitination) remain at the preclinical stage, supported primarily by in vitro proof-of-concept studies. Accordingly, their in vivo efficacy, safety, and effects on bile acid homeostasis require systematic evaluation.

### 3.1. Targeting Phosphorylation: Dynamic Modulation of Membrane Receptor Abundance

Targeting NTCP phosphorylation aims to indirectly modulate its function by intervening at the upstream regulatory hub of membrane localization. This strategy highlights the therapeutic potential of exploiting host intracellular signaling pathways to limit viral entry or regulate substance transport. Recent studies demonstrate that the traditional Chinese medicine formula Guizhi Jiahuangqi Decoction modulates the cAMP/PKC/PKB signaling axis to alter NTCP phosphorylation and membrane positioning, thereby inhibiting HBV entry. Notably, its effects extend beyond NTCP inhibition, disrupting broader stages of the HBV lifecycle [31]. In contrast, tauroursodeoxycholic acid promotes NTCP dephosphorylation and plasma membrane trafficking under hyperosmotic stress via a cAMP-dependent mechanism, thus restoring or enhancing BA uptake [66]. These findings suggest that phosphorylation-based regulatory strategies may offer corrective potential in pathological conditions such as cholestasis.

However, studies on TLC reveal the complex nuances of phosphorylation-mediated regulation. While TLC induces NTCP phosphorylation and internalization through PKC activation, its prolonged inhibitory effects primarily arise from a distinct trans-inhibition mechanism rather than sustained phosphorylation [33]. These observations suggest that phosphorylation events may serve as transient initiators of rapid modulation, with prolonged functional suppression likely driven by more intricate molecular cascades.

Phosphorylation establishes a pervasive and finely tuned regulatory framework for membrane transporters. Beyond NTCP, the membrane abundance, internalization dynamics, and transport efficacy of various solute carrier family transporters are intricately regulated by kinase–phosphatase networks [77,78]. Nevertheless, a significant challenge of this approach is the off-target effects resulting from the pleiotropic nature of these signaling cascades. For example, PKC and PKA interact with numerous substrates in hepatocytes, meaning that non-specific activation or inhibition may lead to unforeseen physiological consequences—an obstacle for current kinase-targeted therapies [79,80].

Therefore, future research should focus on systematically dissecting NTCP-specific phosphorylation regulatory networks and identifying its dedicated regulatory proteins, ultimately enabling the development of precise interventions that finely tune NTCP membrane trafficking while avoiding disruptions to broader cellular signaling pathways.

### 3.2. Targeting Oligomerization: Intervening in the Structural Foundations of Functionality

Modulating NTCP oligomerization represents a key strategy for directly influencing its functionality from a structural perspective. Extensive research has confirmed that NTCP predominantly exists as a dimer on the hepatocyte plasma membrane, with its oligomerization dynamics playing a key role in its intracellular localization and biological properties (as discussed in Section 2.2). For example, troglitazone disrupts dimer assembly through direct interaction with NTCP, effectively reducing HBV entry and impairing BA uptake [16]. Similarly, the synthetic BA derivative SO-145 demonstrates significant antiviral activity by perturbing NTCP oligomerization [17]. These findings support the idea that targeting NTCP oligomerization is a viable approach for functional inhibition.

In addition to disrupting oligomerization directly, steric inhibition of protein–virus interactions offers another potent strategy. The BA derivative INT-767 selectively interacts with the NTCP-binding site within the viral preS1 protein (critical residues: Phe13, Phe14, His17), creating steric hindrance that prevents preS1-NTCP binding and thus suppresses viral entry [71]. Although this strategy does not target oligomerization per se, it highlights the ability of high-affinity ligands to occupy the NTCP functional interface and effectively block NTCP-mediated viral invasion.

Despite the promising potential of these strategies, a significant challenge remains: the lack of atomic-resolution oligomeric structures for NTCP, which impedes structure-based drug design. Currently, the discovery of the most active compounds relies on phenotypic screening. However, innovative approaches have emerged, such as pentacyclic triterpenoid conjugates, which simultaneously target HBV polymerase and NTCP. This dual-targeting strategy provides a novel means of inhibiting viral reverse transcription while obstructing viral entry [81]. These approaches not only offer new avenues for overcoming viral drug resistance but also suggest that, once NTCP’s structural architecture is elucidated, future efforts could engineer single molecular entities capable of modulating oligomerization while integrating additional mechanisms, thereby enhancing antiviral efficacy.

### 3.3. Targeting Ubiquitination: Precisely Obstructing the Viral Endocytosis Step

Protein ubiquitination, a key PTM, is regulated by a cascade involving ubiquitin-activating enzyme (E1), ubiquitin-conjugating enzyme (E2), and E3 ubiquitin ligase, controlling protein stability, molecular interactions, and intracellular trafficking [82,83].

K340 ubiquitination of NTCP plays a pivotal role in viral invasion. Mutation at this site (K340R) leads to increased NTCP membrane abundance and a 220% enhancement in binding affinity for the viral preS1 peptide, yet endocytosis efficiency drops to just 42% [18]. This is further corroborated by the ubiquitination inhibitor TAK-243, which simultaneously boosts BA uptake by approximately 218% while effectively suppressing viral infection. These results indicate that NTCP-mediated HBV endocytosis is heavily dependent on K340 ubiquitination, a modification essential for viral internalization but not for initial viral binding [18].

Building on these mechanistic insights, targeting NTCP ubiquitination opens new avenues for therapeutic intervention, allowing for selective modulation of its physiological and pathological functions. This approach aligns with the concept of precision orchestration via non-degradative ubiquitination signals—signals that mediate functional regulation without triggering proteasomal degradation [84]. This emerging field requires further mechanistic clarification. Given the critical role of E3 ligases in substrate-specific recognition [85], investigating the specific E3 ligases that regulate NTCP—along with their structural bases—will provide the essential framework for precision therapeutics tailored to this pathway.

### 3.4. Other Intervention Strategies: Targeting NTCP Receptor Binding and Viral Competition

In addition to interventions targeting specific PTMs, various compounds exert antiviral effects through direct NTCP interaction or competitive inhibition of viral binding. For example, bulevirtide, a preS1 domain mimetic peptide, competitively binds NTCP’s viral receptor sites, thereby directly preventing HBV entry into host cells [67,68,69,70]. Similarly, cyclosporin A (CsA) and its derivatives interact directly with NTCP via mechanisms independent of their immunosuppressive effects, ultimately inhibiting viral infection [71,72,73,74]. Notably, novel CsA analogs such as SCY450 and SCY995 achieve functional decoupling by preserving NTCP-mediated BA transport while effectively blocking the entry of multiple HBV genotypes [73]. Additionally, BA derivatives like INT-767 selectively recognize the NTCP-binding motif within the viral preS1 domain, creating steric hindrance to disrupt virus–receptor interactions [75]. The feasibility of these strategies is further supported by molecular-level evidence. Mutation of tyrosine at position 146 (Y146) in NTCP completely abolishes binding with the viral preS1 peptide while preserving BA transport function, confirming that the physiological and pathological roles of NTCP can be dissociated at specific amino acid residues [62].

Bulevirtide is currently approved primarily for the treatment of CHD. In the Phase 3 MYR301 trial, 144 weeks of bulevirtide 2 mg monotherapy resulted in an undetectable HDV RNA rate of 29% (14/49) and a combined response rate—defined as undetectable HDV RNA or a ≥2 log_10_ IU/mL decline from baseline together with alanine aminotransferase normalization—of 57% (28/49) in patients with CHD [86]. A Phase 2 study further showed that following 48 weeks of bulevirtide 2 mg monotherapy and a subsequent 24-week off-treatment period, the undetectable HDV RNA rate was 7% (1/15) at week 72 [87]. Notably, treatment was associated with asymptomatic, dose-dependent elevations in total bile acids, which were reversible upon treatment discontinuation [87].

Although these strategies avoid direct structural modifications of NTCP, they significantly expand functional paradigms in NTCP-targeted drug discovery. Currently, HBV entry inhibitors—comprising small molecules and neutralizing antibodies—represent a dynamic frontier in novel therapeutics for chronic hepatitis B, with several candidates advancing into clinical trials [88]. The efficacy of these modalities is attributed to precise mechanistic insights into viral entry dynamics. For instance, research has identified two critical neutralizing epitopes within the preS1 receptor-binding domain, with the linear epitope spanning residues 2–47 of the preS1 N-terminus (preS1 (2–47)) being essential for NTCP engagement, thereby providing discrete targets for the development of high-potency entry inhibitors [89]. In this context, structure-based drug design offers promise for optimizing small-molecule efficacy, while epitope-specific neutralizing antibodies hold therapeutic potential by selectively blocking preS1–NTCP interactions [90]. Moving forward, the key challenge will be to balance robust antiviral efficacy with the preservation of hepatic BA metabolic homeostasis, a key factor for the clinical translation of these strategies.

## 4. Summary and Outlook

The diverse PTMs of NTCP create a dynamic, reversible regulatory network that integrates its dual functions in BA transport and HBV/HDV receptor interaction. Targeting these PTMs—such as phosphorylation, oligomerization, and ubiquitination—has emerged as a promising strategy for the development of novel antiviral drugs. This approach, which modulates upstream functions, bypasses the limitations of conventional direct-binding inhibitors. When combined with established nucleos(t)ide analogs, this strategy holds transformative potential by complementing viral entry blockade and replication suppression, ultimately offering new avenues toward improved virological responses and potentially a functional cure [91,92].

Beyond antiviral applications, NTCP modulation shows expansive translational promise for a range of liver diseases. Recent studies suggest that targeting NTCP could offer therapeutic benefits for conditions like non-alcoholic fatty liver disease, hepatic fibrosis, primary biliary cholangitis, and hepatocellular carcinoma [93]. This rationale is rooted in NTCP’s critical role as the primary portal for hepatic BA uptake, with its dysfunction being a hallmark feature of various cholestatic liver disorders [94,95]. Therefore, therapeutic strategies that precisely regulate NTCP’s membrane localization or transport kinetics could offer innovative treatments tailored to specific cholestatic syndromes. This trajectory exemplifies the continuum from benchside discovery to bedside application, with NTCP serving as a versatile target—repurposed for antiviral prophylaxis, hepatoprotection, and choleretic enhancement.

However, targeting PTMs poses significant challenges due to the broad signaling cascades involved, which affect multiple cellular processes, creating a high risk of off-target effects. Non-specific inhibition of kinases like PKC, for example, could disrupt a wide range of physiological functions, including hepatocyte cytoprotection and tumorigenesis [96,97], while indiscriminate modulation of E3 ubiquitin ligases may undermine tumor-suppressive pathways, such as those mediated by FBXO7 [98]. Consequently, key breakthroughs will depend on in-depth studies to identify the kinases or E3 ligases specifically modulating NTCP functionality. This precision would allow for the development of high-selectivity agents with reduced off-target risks and improved safety profiles. Furthermore, it should be noted that most of the evidence reviewed here is derived from in vitro or cell-based models, whereas in vivo and clinical validation of PTM-targeting strategies remains limited, representing a critical gap that warrants further investigation. Moreover, several fundamental questions remain entirely unexplored, including how different PTMs crosstalk (e.g., phosphorylation influencing ubiquitination), the temporal sequencing of PTM events, and whether combinatorial PTM codes exist on NTCP. Addressing these questions will be essential for a comprehensive understanding of NTCP regulation.

Although “functional decoupling” inhibitors, such as the CsA derivative SCY450, offer an innovative approach to block viral entry while preserving BA uptake [73], their design and optimization present substantial challenges. One critical issue is the potential disruption of BA homeostasis with prolonged use. Given the essential role of BAs in lipid absorption and metabolic regulation, such disruptions could lead to metabolic complications, including fat malabsorption [99]. Therefore, early drug discovery efforts should focus on in vitro models that are particularly sensitive to BA imbalances, enabling effective risk assessment [100] and prioritizing dynamic profiling of BA spectra alongside antiviral potency. In this context, ubiquitination-targeting (e.g., TAK-243) and HBV preS1-binding (e.g., INT-767) strategies emerge as the most promising approaches for achieving functional decoupling, thereby warranting prioritized in vivo validation.

In summary, this review highlights the dual significance of investigating the NTCP PTM network—both for antiviral innovation and metabolic regulation. It provides detailed mechanistic insights into NTCP as a promising antiviral target while also advancing our understanding of its role in hepatic BA homeostasis. Future research will rely on the integration of multidisciplinary technologies to uncover NTCP modification patterns, identify key targets, and develop precision therapeutics. These next-generation therapies have the potential to revolutionize chronic hepatitis B treatments and extend their benefits to cholestatic liver diseases and other related disorders.

## Figures and Tables

**Table 1:** Strategies targeting NTCP and its regulatory pathways for antiviral interventions and bile acid homeostasis modulation.

Category	Drug/Compound Name	Mode of Action	Primary Mechanism of Action	Effect on HBV/HDV Entry	Effect on BA Transport	References
Oligomerization	Troglitazone	Disruption of NTCP oligomerization	Prevents formation of functional multimers, reducing viral entry	inhibited	impaired	[16]
	Bile Acid Derivative, SO-145	Disruption of NTCP oligomerization	Directly interacts with NTCP to prevent oligomerization	inhibited	Not reported	[17]
Ubiquitination	TAK-243	Inhibition of NTCP ubiquitination	Blocks K340 ubiquitination, preventing endocytosis of HBV-NTCP complex	inhibited	minimally affected	[18]
Phosphorylation	Guizhi Jia Huangqi Decoction	Modulation of phosphorylation signaling	Alters NTCP phosphorylation and membrane localization via cAMP/PKC/PKB axis	inhibited	Not reported	[31]
	Bile Acid Derivative, Tauroursodeoxycholic Acid	Modulation of phosphorylation signaling	Promotes NTCP dephosphorylation and membrane reinsertion under stress	Not reported	restored/enhanced	[66]
Other modes	CDC42	Membrane trafficking regulation	Promotes NTCP plasma membrane localization and macropinocytosis	enhanced	not reported	[20]
	Bile Acid Derivative, Taurolithocholate	Functional inhibition (trans-inhibition)	Prolonged suppression of NTCP transport activity via trans-inhibition	inhibited	inhibited	[33]
	Bulevirtide (Myrcludex B)	Receptor binding competition	Competes for NTCP viral binding sites as preS1 mimetic	inhibited	competitively inhibited	[67,68,69,70]
	Cyclosporin A and Its Derivatives	Receptor binding (conformational modulation)	Binds NTCP to suppress functionality	inhibited	inhibited	[71,72,73,74]
	Bile Acid Derivative, INT-767	Binds to the HBV preS1 binding domain	Selectively targets NTCP-binding motif (Phe13, Phe14, His17) in viral preS1, blocking virus-receptor interaction	inhibited	no direct effect	[75]
	Dimeric Bile Acid Derivatives	Receptor binding (conformational modulation)	Engages NTCP to induce conformational changes that attenuate its functional activity.	inhibited	inhibited	[76]

## Data Availability

No new data were created or analyzed in this study.

## Abbreviations

NTCP, Na^+^/taurocholate cotransporting polypeptide; PTMs, post-translational modifications; BA, bile acids; HBV, hepatitis B virus; HDV, hepatitis D virus; ASBT, apical sodium-dependent bile acid transporter; cAMP, cyclic adenosine monophosphate; PP2B, protein phosphatase 2B; PI3K, phosphoinositide 3-kinase; PKA, protein kinase A; PKB, protein kinase B; PKC, protein kinase C; cPKC, conventional protein kinase C; UPS, ubiquitin–proteasome system; ERAD, endoplasmic reticulum-associated protein degradation; HSPGs, heparan sulfate proteoglycans; EGFR, epidermal growth factor receptor; cccDNA, covalently closed circular DNA; CsA, cyclosporin A; CHD, chronic hepatitis D.
